# Immobilization of KR-12 on a Titanium Alloy Surface Using Linking Arms Improves Antimicrobial Activity and Supports Osteoblast Cytocompatibility

**DOI:** 10.1021/acsabm.4c01731

**Published:** 2025-03-28

**Authors:** Mohadeseh Zare, Laura Colomina Alfaro, Antonella Bandiera, Esra Cansever Mutlu, David Grossin, Fernando Albericio, Sarah A. Kuehne, Zubair Ahmed, Artemis Stamboulis

**Affiliations:** † Biomaterials Research Group, School of Metallurgy and Materials, University of Birmingham, Edgbaston, Birmingham B15 2TT, U.K.; ‡ Department of Life Sciences, University of Trieste, via L. Giorgieri 1, Trieste 34127, Italy; § CIRIMAT, Toulouse INP, Université Toulouse 3 Paul Sabatier, CNRS, Université de Toulouse, 4 Allée Emile Monso, BP44362, 31030 Toulouse, Cedex 4, France; ∥ School of Chemistry and Physics, University of KwaZulu-Natal, Durban 4000, South Africa; ⊥ School of Science and Technology, Nottingham Trent University, Nottingham NG11 8NS, U.K.; # Neuroscience and Ophthalmology, Department of Inflammation and Ageing, School of Infection, Inflammation and Ageing, College of Medicine and Health, University of Birmingham, Edgbaston, Birmingham B15 2TT, U.K.

**Keywords:** antimicrobial peptide, Human-Elastin Like Polypeptide, KR-12, enzyme-responsive coating, osteoblast promotion, bacterial growth inhibition

## Abstract

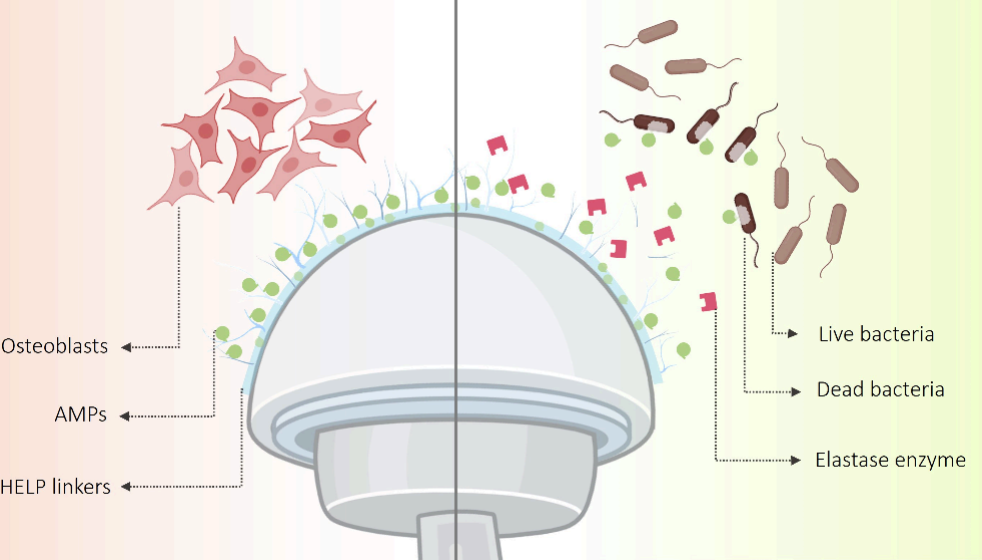

Implant-associated infections pose significant challenges due to bacterial resistance to antibiotics. Recent research highlights the potential of immobilizing antimicrobial peptides (AMPs) onto implants as an alternative to conventional antibiotics for the prevention of bacterial infection. While various AMP immobilization methodologies have been investigated, they lack responsiveness to biological cues. This study proposes an enzyme-responsive antimicrobial coating for orthopedic devices using KR-12, an AMP derived from Cathelicidin LL-37, coupled with the Human Elastin-Like Polypeptide (HELP) as a biomimetic and stimuli-responsive linker, while mimicking the extracellular matrix (ECM). During implantation, these customized interfaces encounter the innate immune response triggering elastase release, which degrades HELP biopolymers, enabling the controlled release of KR-12. After coupling KR-12 with HELP to titanium surfaces, the antimicrobial activity against four pathogenic bacterial strains (*Staphylococcus aureus*, *Staphylococcus epidermidis*, *Escherichia coli*, and *Pseudomonas aeruginosa*) was assessed, revealing an inhibition ratio of bacterial adhesion and colonization exceeding 92% for all tested strains, compared with surfaces functionalized with KR-12 only. It is thought that the enhanced antimicrobial activity was due to the improved mobility of KR-12 when coupled with HELP. Furthermore, the prepared coatings boosted the adhesion and proliferation of human osteoblasts, confirming the cytocompatibility. These findings suggest the potential for smart coatings that combine the antimicrobial functions of AMPs with HELP’s biological properties for use in a variety of settings, including medical devices.

## 1 Introduction

The implantation of medical devices carries a notable risk of nosocomial pathogens colonizing and forming biofilms on device surfaces, subsequently leading to infections with prolonged healing periods. According to the concept of the “race for the surface”, proposing a competition between host and bacterial cells for colonization on the implant surface, the likelihood of bacterial attachment decreases if the host cells establish colonization on the implant surface first, and vice versa.^1^ Upon implant placement, the surface becomes coated with a protein film, providing potential receptors for bacterial adhesive ligands, thus promoting further colonization. Biofilms, once formed, serve as effective shields for microorganisms against host immune responses and antibiotic treatments.^2^

Titanium, renowned for its mechanical strength, chemical inertness, and compatibility with biological systems, remains the predominant material choice in orthopedic implants, bone surgery, and dental applications, aiming to improve human tissue healing.^3^ However, despite its exceptional characteristics, bacterial colonization on titanium surfaces remains a significant factor contributing to implant failure, often requiring revision and introducing complexities to patient treatment and recovery processes.^4^

To effectively inhibit bacterial biofilm formation, it is essential to modify surfaces with antimicrobial coatings to combine antifouling methods to deter bacterial adhesion and colonization while enhancing biocompatibility by reducing potential toxicity to human cells.^5^ Recent advancements in antimicrobial coatings aim to develop multifaceted structures with controllable toxicity and bioengineering capabilities. These novel coatings are designed to achieve a balance between effective pathogen elimination and compatibility with host tissues, minimizing cytotoxic effects.^6^ Innovations include incorporating nanostructured materials, stimuli-responsive elements, and multifunctional agents, such as nanoparticles or antimicrobial peptides (AMPs), which provide controlled release or activation in response to specific environments.^7,8^ This dual functionality addresses the challenges of preventing infections while supporting tissue regeneration and integration by promoting cellular adhesion, proliferation, and differentiation.^9,10^ AMPs, which are naturally found in various organisms, possess distinct structural features characterized by cationic and amphiphilic domains. These features are crucial for disrupting bacterial cell membranes. Unlike traditional antibiotics, AMPs have demonstrated a broad spectrum of activity against bacteria, fungi, and viruses while also avoiding the induction of bacterial resistance. This resistance avoidance stems from their ability to permeate the cytoplasmic membrane without triggering typical resistance mechanisms.^11^

Amidst a diverse array of AMPs that have been documented, the human cathelicidin peptide LL-37 emerges as a noteworthy antimicrobial agent renowned for its multifaceted immunomodulatory capabilities.^12^ One notable fragment of LL-37 is the cationic KR-12; the shorter length, reduced cytotoxicity, and cost-effectiveness of KR-12 make it an attractive option for various biomedical applications, including surface immobilization on materials used in wound dressings, urinary catheters, and implants.^13^ KR-12 interacts favorably with bacterial membranes due to its α-helical conformation and positive charge while also minimizing cytotoxic effects. Structural studies suggest that AMPs like KR-12 function by creating nanometric pores in cell membranes or inducing membrane micellization.^14^ Moreover, prior research has shown that controlled concentrations of KR-12 promote osteogenic differentiation in human-bone-marrow-derived mesenchymal stem cells, in addition to its antibacterial properties.^15,16^

Two primary approaches, physical adsorption and covalent immobilization, have been explored for immobilizing AMPs on titanium implants.^17^ The simpler method, involving the physical adsorption of AMPs onto the titanium surface, is less stable compared to covalent immobilization, leading to rapid leaching of AMPs and a subsequent reduction in antimicrobial activity over time.^17,18^ In contrast, covalent immobilization establishes a chemical bond between the AMP and the titanium surface, ensuring long-term stability and preserving AMP activity on the implant surface.^19^ However, a drawback of this method is that it reduces the mobility of the peptide and can cause steric hindrance. To address this limitation, a flexible linker between the AMP and the titanium surface can be introduced as a solution. The linkers provide flexibility to AMPs, increasing their exposure and facilitating better mobility and penetration of bacteria, thereby enhancing antimicrobial efficacy.^20^

Although covalent immobilization of AMPs using linkers ensures high antimicrobial efficiency, the increased concentration of antimicrobial agents on the surface may compromise the biocompatibility, angiogenic activity, and osteogenic activity of the implant, consequently delaying sufficient osseointegration.^21^ To address these critical issues, there is a demand to explore novel antimicrobial coatings capable of selectively targeting bacteria under pathological conditions.^22^ Therefore, developing statically versatile implant coatings would significantly enhance their applicability, necessitating the design of a coating capable of responding to fluctuations in the pH or enzymatic activity.^21^ The findings of recent studies emphasized the significance of biomaterial coatings that respond to pathogen microenvironments inhibiting the contact between AMPs and normal cells and thereby decreasing their toxicity.^23,24^ Such an approach was followed by Suo et al.,^23^ who reported that AMP–hyaluronic acid hybrid hydrogels exhibit excellent broad-spectrum antibacterial activity, showing on-demand release of AMPs specifically in acidic environments and expediting wound healing in infected mice. These findings suggest a promising avenue for the development of biomaterial coatings that could effectively manage chronic infected wounds by harnessing the innate properties of AMPs and responsive hydrogel systems.

The ideal antimicrobial coatings should exhibit efficacy against diverse pathogens, biocompatibility with host tissues, long-lasting activity, physiological stability, minimal cytotoxicity, resistance to biofilm formation, ease of application, and cost-effectiveness for medical deployment.^25^ To meet these criteria for the anticipated antimicrobial coating, an ideal linker for immobilizing AMPs on titanium implants could be extracellular matrix (ECM)-inspired polymers, such as Human Elastin-Like Polypeptide (HELP).^26^ HELP is a well-described recombinant biopolymer comprising sequences resembling natural human tropoelastin, offering enhanced biocompatibility, improved cell adhesion, and increased anchoring sites for effective AMP immobilization.^27^ Moreover, HELP can be degraded by physiological pathways, mitigating the risk of harmful byproducts.^28^ Previous studies have assessed the proteolytic susceptibility of HELP matrixes, demonstrating their potential for enzymatic-triggered release of model compounds.^29,30^ The elastolytic activity, present in both prokaryotic and eukaryotic organisms, is associated with various pathological conditions such as pulmonary emphysema, cystic fibrosis, infections, inflammation, chronic wounds, and atherosclerosis.^31,32^ Building on this concept, HELP polypeptides show promise as proteolytic stimuli-responsive systems for controlling the release of AMPs.^28^

In this study, we developed an ECM-mimicking system based on HELPs to tether AMPs to titanium implant devices. We utilized enzyme-mediated release, taking advantage of physiologically normal inflammatory reactions or remodeling processes, which are most active during tissue healing and bone formation after surgical implantation.^30^ Initially, HELP biopolymer was immobilized onto titanium disks as linkers. Subsequently, the AMP KR-12 was attached covalently at an optimized immobilization density to maximize the cell viability and antimicrobial activity. The antimicrobial activity of the coating against various pathogenic bacteria was investigated, and the cytocompatibility of the coating was assessed by using human osteoblast bone cells.

## 2 Experimental Methods

### 2.1 Sample Preparation

**Figure 1:**
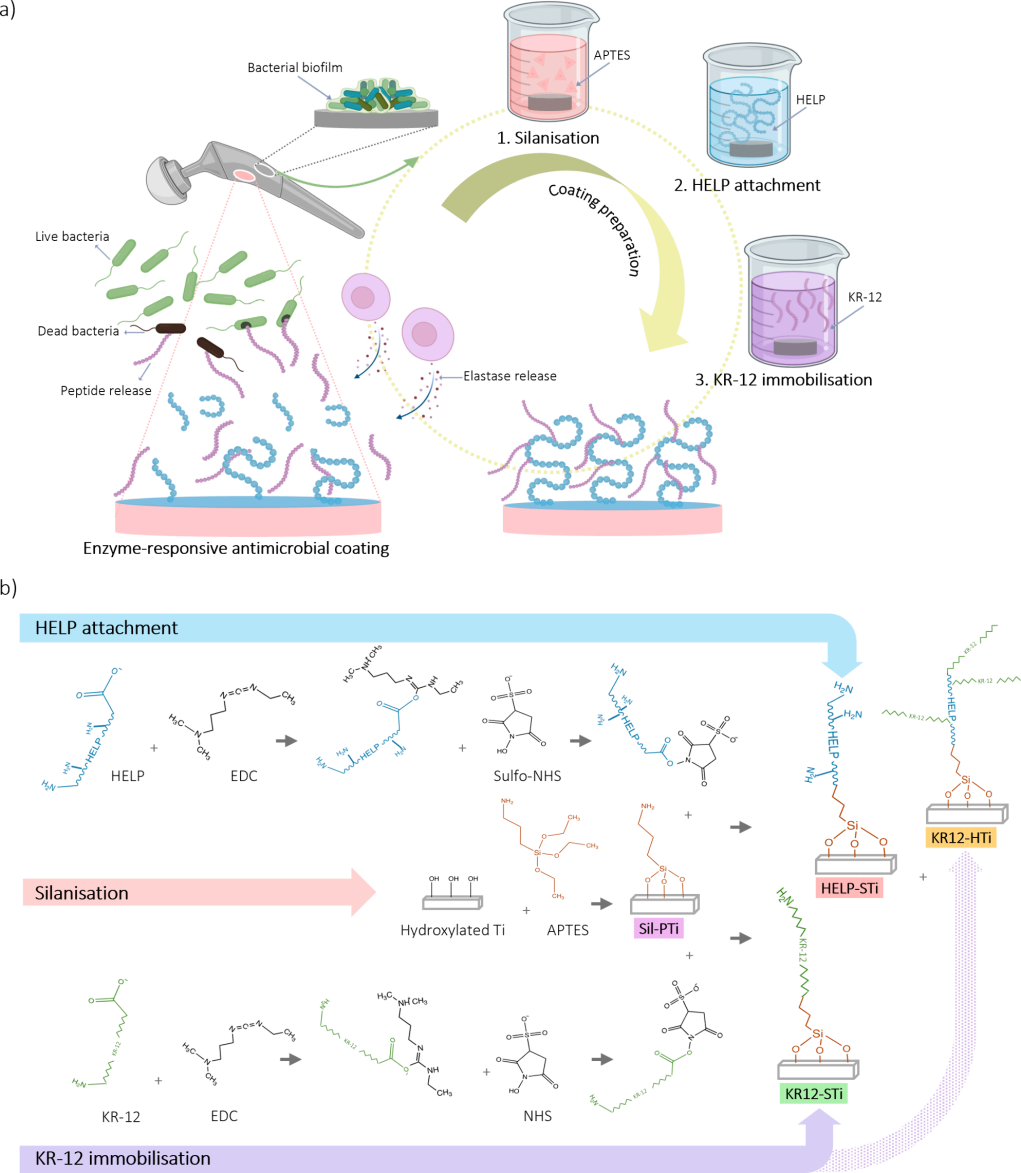
Schematic representation of (a) a step-by-step procedure for silanization, HELP attachment, and KR-12 immobilization to prepare an enzyme-responsive antimicrobial coating for titanium implant surfaces and (b) chemical reactions that occurred in each step at the interface of the titanium surface and the reagents. The diagram is not drawn to scale, and symbols are just utilized to elucidate the underlying concepts. APTES = (3-aminopropyl)triethoxysilane, HELP = Human Elastin-Like Polypeptide, EDC = 1-ethyl-3-[3-(dimethylamino)propyl]carbodiimide hydrochloride, and sulfo-NHS = sulfo-*N*-hydroxysuccinimide.

The immobilization of peptides on titanium substrates involved a systematic three-step process. Prior to initiating the process, the production and purification of HELP was carried out. To immobilize the peptides using HELP linkers on the titanium substrates, first, hydroxylation and silanization were carried out, followed by attachment of the HELP linker and subsequent peptide immobilization. Each stage of this sequential procedure is precisely outlined in the following sections. The processing scheme is summarized in Figure 1. Table 1 provides the description of sample names used in the experimental work.

**Table 1:** Description of Sample Names Indicating Different Surface Modifications

Sample Name	Description
PTi	Polished titanium alloy Ti6Al4 V
Sil-PTi	Silanized titanium surface
KR12-STi	KR-12 immobilized on Sil-PTi
HELP-STi	HELP attached on Sil-PTi
KR12-HTi	KR-12 immobilized on HELP-attached Sil-PTi

#### 2.1.1 Silanization of Titanium Substrates

Titanium disks, 10 mm in diameter and 2 mm in thickness, served as representative models for the prevalent materials found in dental and orthopedic implants (commercially pure Medical grade 5 Ti6Al4 V, William Gregor Ltd., East Grinstead, U.K.). The preparation process started with grinding of the titanium disks consecutively with 800, 1200, and 2500 grit Struers silicon carbide/abrasive papers using Struers Metallographic equipment. The disks were then polished to a mirror finish using a 0.05-μm-particle-size colloidal silica, according to Bruker’s protocols. To ensure cleanliness, the polished disks were sonicated for 30 min in ethanol, followed by a 30 min sonication in deionized (DI) water. The titanium disks were then air-dried at room temperature and stored in a desiccator until required.

To facilitate the silanization process, a thorough 2 h etching process using an oxidizing solution (50:50 v/v, 98% H_2_SO_4_/30% H_2_O_2_) was carried out, introducing hydroxyl groups on the surfaces. The etched surfaces were washed twice in DI water and ethanol with 30 min sonication intervals. Silanization, a common method for immobilizing biomolecules on inorganic surfaces, was then performed, as described previously.^33^ An organic silane, (3-aminopropyl)triethoxysilane (APTES) (ThermoFisher Scientific, Birmingham, U.K.), was used as the intermediate linker, enhancing the adhesion process. The hydroxylated titanium (TiOH) substrates were immersed in a 10% (v/v) APTES solution in toluene (purity ≥ 99.5%, Sigma-Aldrich, Poole, U.K.) at 80 °C for 4 h with continuous agitation to introduce functional amino groups onto the titanium surfaces. Each batch of the silanization reaction consisted of 10 samples in a total volume of 10 mL of solution. After the silanization reaction, the surfaces were sonicated following two 10 min sonication cycles in each of the following solvents: pure toluene, ethanol, and water.

#### 2.1.2 Production and Purification of the HELP Biopolymer

The expression and purification of recombinant HELP biopolymers were performed in standard conditions, as described in the literature.^34,35^ Briefly, recombinant clones of the *Escherichia coli* C3037I strain (New England Biolabs, Ipswich, MA) transformed with the plasmid carrying HELP DNA sequences were grown in a Luria–Bertani (LB) medium, supplemented with 50 μg/mL ampicillin and 70 μg/mL chloramphenicol. An overnight starter culture was used to seed the expression culture in Terrific Broth, which was carried out at 37 °C under constant agitation until a turbidity of approximately OD_600_ = 1 was reached. The expression of HELP was induced by adding 0.1 mM isopropyl β-d-1-thiogalactopyranoside. Growth was continued for an additional 5 h, after which bacterial cells were collected by centrifugation at 8000 rpm for 20 min at 10 °C using a Beckman Coulter J-26 XP (High Wycombe, U.K.).

The pellets were suspended in 400 mL of an extraction solution containing 50 mM Tris/HCl (pH 8), 250 mM NaCl, 0,1 mM EDTA, 0,1% Triton X-100, and 1 mM phenylmethanesulfonyl fluoride and disrupted using a high-pressure homogenizer (Panda NS1001L, GEA Niro Soavi, Italy). Then, 2-mercaptoethanol was added to 20 mM, and after the bacterial lysate was cooled on ice, the mixture was centrifuged at 10000 rpm for 30 min at 8 °C (Beckman Coulter J-26 XP). The cellular debris was discarded, and the supernatant was further processed to purify the HELP biopolymer following a well-established procedure known as inverse transition cycling (ITC), which is based on the thermoresponsive properties of the elastin domain.^36^ Briefly, the biopolymer was precipitated by adding NaCl to the supernatant to a final concentration of 1.5 M at 37 °C. The aggregated part was separated from the solution by centrifugation at 7000 rpm for 30 min at 37 °C. The pellet was redissolved in cold water, and any nonsoluble components were removed through cold centrifugation. In total, three ITC cycles were performed to ensure the desired degree of purification. The material was lyophilized for long-term storage and further use. The details of the HELP biopolymer sequences are provided in the Supporting Information.

#### 2.1.3 HELP Attachment

To enable the attachment of HELP as a linker onto titanium surfaces, the process involved the selective activation of the carboxyl group in HELP using 1-ethyl-3-[3-(dimethylamino)propyl]carbodiimide hydrochloride (EDC, ThermoFisher Scientific), resulting in the formation of highly reactive *O*-acylisourea. This intermediate complex subsequently reacted with the primary amine groups present on the titanium surface using sulfo-*N*-hydroxysuccinimide (sulfo-NHS, ThermoFisher Scientific) as an activating agent, as described in the literature.^37^

The initial step involved preparing a 1 mg/mL HELP solution in 2-(*N*-morpholino)ethanesulfonic acid (MES) buffer (0.1 M, pH 6.0). Amino-functionalized substrates (Sil-PTi; Table 1) were then immersed in a HELP solution for 1 h, followed by immersion in a solution of EDC (23 mg/L) and the sequential addition of sulfo-NHS (77 mg/L) in a dropwise manner. Each batch of the reaction included 10 samples within a total volume of 10 mL. The grafted samples, HELP-STi, were successively washed with DI water and ethanol using a vortex to remove noncovalently bound reactants before drying and storing in a desiccator.

#### 2.1.4 Covalent Immobilization of KR-12

Following the attachment of HELP, a similar EDC/NHS protocol was employed to immobilize KR-12 (H-KRIVQRIKDFLR-OH) on the amine groups of HELP, labeled as KR12-HTi. An identical procedure was employed to immobilize KR-12 (BOCSCI Inc., NY, USA) directly onto the silanized surface of Sil-PTi, labeled as KR12-STi. To immobilize the peptides, the carboxylic groups of the peptides were activated with EDC and subsequently reacted with the primary amine groups of the silanized surface of Sil-PTi or the HELP biopolymer on the surface of HELP-STi. Following the protocol outlined in the previous section, the substrates were incubated in a 1 mg/mL solution of KR-12 in MES buffer. After 1 h of incubation, EDC and sulfo-NHS solutions were added dropwise in sequence to facilitate the covalent binding of the KR-12 peptide. This step-by-step procedure ensured a consistent and effective process for immobilization of the KR-12 peptide onto the silanized surfaces and HELP-modified surfaces. In both procedures, each batch consisted of 10 samples processed in a total volume of 10 mL. Two wash cycles with DI water and ethanol, employing a vortex for thorough cleansing, were performed to remove any noncovalently bound peptides.

To precisely quantify the immobilized peptides on the surfaces, a fluorenylmethyloxycarbonyl (Fmoc)-labeled KR-12 [Fmoc-KRIVQRIKDFLR-OH] was used, following the protocol mentioned above. The peptide percentage used for KR-12 immobilization was selected and evaluated based on the minimum inhibitory concentration (MIC) of KR-12. KR-12 and modified versions of KR-12 have been synthesized in our laboratory by solid-phase peptide synthesis and fully characterized. The MIC values for KR-12 in this study were measured at 2, 2, and 8 μM for *E. coli*, *Pseudomonas aeruginosa*, and *Staphylococcus aureus*, respectively, consistently close to reported values from previous studies.^38,39^

However, the antimicrobial efficacy observed with the immobilized peptide on surfaces differed significantly from the expected results based on MIC. The reason behind this is that AMPs often exhibit different mechanisms and efficacies compared to their soluble counterparts^40^ (see also Results and Discussion sections 3.1, 3.2, and 3.4). When peptides are attached to a surface, their mobility is restricted. This can lead to more rigid and extended conformations, which may affect their interactions with other molecules. In addition, the immobilization process can influence the peptide’s orientation, accessibility, and interaction with bacterial membranes, leading to variations in antimicrobial activity compared to the free form of the peptide.^41^ Consequently, we incrementally increased peptide concentrations until the optimum antimicrobial activity was achieved. This approach ensured the highest antimicrobial activity, while optimized concentrations conducive to osteogenic differentiation and promotion were determined.

### 2.2 Surface Characterization

#### 2.2.1 Scanning Electron Microscopy (SEM)

At each stage of sample preparation, SEM was carried out to assess the influence of individual steps on the surface morphology using a JEOL 6060 with an Oxford Inca energy-dispersive spectrometer. Before the examination, the samples were gold (Au) sputter-coated using a Polaron Emitech SC7640 sputter coater. Each sample was then subjected to a comprehensive imaging process, capturing five images at a working distance of 9 mm and a voltage of 20 kV.

#### 2.2.2 Attenuated-Total-Reflectance Fourier Transform Infrared (ATR-FTIR)

To confirm the chemical reaction mechanisms, the presence of functional groups was assessed through ATR-FTIR spectral analysis before and after the chemical reactions using a Nicolet 6700 FTIR machine (ThermoFisher Scientific) and Omnic 8 software suite (ThermoFisher Scientific) in the range of 4000–400 cm^–1^. The spectra were acquired from an average of 64 scans/background at a spectral resolution of 2 cm^–1^.

#### 2.2.3 Atomic Force Microscopy (AFM)

The surface topography and roughness changes of HELP-attached and peptide-attached samples were analyzed in the tapping mode at room temperature using Bruker Multimode 8 and Pt-coated silicon cantilevers provided by Bruker (SCM-PIT). Scans were taken at random sites in 20 μm^2^ using a 5 mm scan head at a scan rate of 0.1 Hz for all measurements. The pictures were digitally processed by NanoScope Analysis 1.7 software, and the mean roughnesses (*R*_a_) were measured.

#### 2.2.4 X-ray Photoelectron Spectroscopy (XPS)

The surface elemental compositions of PTi and Sil-PTi were measured using a Thermo NEXSA XPS instrument equipped with a monochromated Al Kα X-ray source (1486.7 eV). The system included a spherical sector analyzer, three multichannel resistive plates, and 128-channel delay line detectors. Data acquisition occurred at 19.2 W and an X-ray beam with a size of 400 × 200 μm. Survey scans were performed at a pass energy of 200 eV, and high-resolution scans used a pass energy of 40 eV. Electronic charge neutralization was managed via a dual-beam low-energy electron/ion source (ThermoFisher Scientific FG-03). Measurements were taken at a pressure below 10^–8^ Torr and a room temperature of 294 K. Data processing was completed using CasaXPS v2.3.20PR1.0 with peaks fitted using Shirley background subtraction prior to component analysis. Binding energies were calibrated with the C 1s signal at 284.8 eV and a minimum of three different samples were analyzed for each group.

#### 2.2.5 Water Contact Angle (WCA) Measurement

Surface wettability, determined through static WCA measurements, is a strong indicator of the surface’s hydrophilic/hydrophobic properties that influence the cells attachment. Static WCAs were measured at room temperature, analyzing the profile of sessile drops. A 5 μL droplet of DI water was deposited on the sample surface by using a Hamilton syringe set on a Kruss DSA100 apparatus (Hamburg, Germany) equipped with a CCD camera and an image analysis processor. The WCAs were calculated by using the built-in CAM 100 software. The test was performed in triplicate. Four droplets were measured at different locations on each sample and three different samples in each group. A static picture was taken within 3 s after droplet deposition, and angles were measured on the static picture. The reported values are the averages of these nine measurements for each type of surface.

### 2.3 Concentration of Immobilized KR-12

The quantification of KR-12 loading on the surface involved estimating the number of Fmoc groups by measuring the maximum ultraviolet (UV) absorption at 301 nm. The determination of Fmoc group loading at the surface enabled calculation of the peptides because each peptide contained one Fmoc (Fmoc-KRIVQRIKDFLR-OH). Fmoc groups attached to the peptides were readily cleaved using 20% piperidine in dimethylformamide (DMF), a common reaction mechanism during solid-phase peptide synthesis employing the Fmoc/*tert*-butanol (t-Bu) method. The presence of piperidine induces the formation of dibenzofulvene–piperidine adducts via a Michael-type addition, which are soluble in reaction solvents, enabling spectrophotometric measurement based on the Beer–Lambert law.^42^ A multipoint standardization method (calibration curve) was established to measure the solution’s UV-light absorption ability at a specific wavelength. The calibration points were prepared and validated on the same day of the analysis to ensure accuracy.

For the calibration curve preparation, Fmoc-KR12 stock solutions (1 mg/mL = 637 μM) were prepared in 20% (v/v) piperidine in DMF. Both solvents were provided by Sigma-Aldrich. Fmoc removal reactions were carried out for 30 min at room temperature. Subsequently, 5.0 mL of the stock solution was diluted to 50.0 mL with 20% (v/v) piperidine in DMF. Dibenzofulvene-based solutions of varying concentrations (ranging from 10 to 310 μM in 30 μM intervals) were prepared using a dilution technique. These solutions were prepared in triplicate and measured at 301 nm, where absorption was solely due to the dibenzofulvene-based adduct. Then, a calibration curve of the dibenzofulvene-based adduct concentration versus absorbance was constructed.

For analysis, each peptide-immobilized titanium surface was immersed in a 20% (v/v) piperidine solution in DMF, and Fmoc removal reactions were conducted for 30 min at room temperature. Subsequently, the absorbance of each solution was measured at 301 nm (*n* = 3) and correlated to the calibration curve.

### 2.4 KR-12 Release Triggered by Elastase Degradation of HELP

As described in previous studies, the HELP moiety has been reported to be susceptible to degradation in the presence of elastase.^35^ Elastases are a family of proteases with broad specificity, with elastin being the main target, because they cleave proteins at the carboxyl side of small hydrophobic amino acids.^43^ For the release of KR-12 from the KR12-HTi and KR12-STi disks, the samples were soaked in 50 mM Tris/HCl (pH 7.5), 1 mM CaCl_2_ buffer, and 50 μL of solution of the elastase enzyme from porcine pancreas (Sigma-Aldrich, #E7885; 50 μg/mL final concentration). Then, they were incubated with the elastase solution at 37 °C for specific time points of 2, 4, 6, 12, and 24 h in a final reaction volume of 500 μL. In parallel, the same conditions were adopted to set up control reactions for KR12-HTi and KR12-STi without adding the enzyme. To assess the amount of KR-12 remaining after elastase-mediated degradation, the samples were removed from the reaction solution and washed gently with DMF at fixed times. Then, piperidine/DMF cleavage was utilized by the protocol described above. The remaining amount subtracted from the amount of measured immobilized peptides provided the released amount of peptide during the enzyme degradation assay.

### 2.5 Antimicrobial Activity Assay

Orthopedic implant infections are caused most often by *S. aureus*, *S. epidermidis*, *E. coli*, and *P. aeruginosa*, which can be acquired during surgery or subsequently through a hematogenous route.^44−46^

To assay the antimicrobial activities of the prepared coatings, two Gram-positive bacteria, *S. aureus* (SH1000) and *S. epidermidis* (ATCC 12228), and two Gram-negative bacteria, *E. coli* (ATCC 25922) and *P. aeruginosa* (PAO1), were grown on Mueller–Hinton (MH) agar plates using the Streak Plate method. Then, a colony of each strain was cultured in MH broth at 37 °C overnight. To count the number of colony-forming units (CFU) in a bacterial suspension, serial dilutions of the bacterial culture were prepared in a 96-well plate and cultured on MH agar plates based on the Miles and Misra method.^47^ The bacterial suspension was adjusted to a concentration of 1 × 10^5^ CFU/mL in MH broth for further analysis.

The coated titanium samples and polished substrates (control samples) were sterilized in 1 mL of 70% ethanol for 5 min at room temperature and then washed with sterilized DI water in a sterile environment. This process was repeated three times. Then, 20 μL of the bacterial suspension was added to the samples, followed by incubation for 24 h at 37 °C. Bacterial growth inhibition, confocal laser scanning microscopy, and SEM were used to characterize the bacterial colonization for each group of coatings. All work was carried out in a class II ventilated flow cabinet, and all tools were autoclaved or sterilized with 70% ethanol before use.

#### 2.5.1 Bacterial Growth Inhibition

The surface antimicrobial activity was tested against *S. aureus*, *S. epidermidis*, *E. coli*, and *P. aeruginosa* using a modified direct contact test (DCT) protocol. For each sample, a sterile Petri dish was prepared with a round filter paper and saturated with phosphate-buffered saline (PBS) to maintain humidity and avoid evaporation of the bacterial suspension. A sterile microscope slide was positioned on the filter paper, and the sample (10 × 10 mm^2^) was kept on the slide. Then, 20 μL of the overnight bacterial suspension (containing 10^5^ bacteria/mL) was pipetted onto the sample surface and a coverslip (20 × 20 mm^2^) was carefully positioned on top to spread the inoculum over the entire disk surface. Petri dishes containing the disks and bacteria were incubated at 37 °C for 24 h.

After incubation, the substrates covered with the bacterial suspension were moved to a 10 mL sterile tube and, after the addition of 5 mL of MH, were vortexed for 5 min to detach adherent bacteria. Because the titanium substrates are nonporous and nonabsorbent, a 5 min process was efficient in removing the adhered bacteria. This has been attested by SEM studies, before and after vortexing. A total of 200 μL of the bacterial suspension was collected, and 10-fold serial dilutions of the suspension were prepared to count the bacterial colonies on MH agar plates using the Miles and Misra method. After incubation at 37 °C for 20 h, the number of colonies on each sample was counted.

The bacterial growth inhibition ratio was calculated by measuring the CFU of the control sample, PTi, (denoted as B1), and each coated titanium (denoted as B2) by using the following equation:
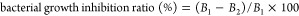
For each sample type, measurements were conducted for three independent experiments; each experiment was performed in triplicate, and the percentage of bacterial growth inhibition was averaged over the three experiments.

#### 2.5.2 Bacteria Morphology Assay

After incubation with 20 μL of bacterial suspension (as described previously), the samples were immersed in 2.5% EM-grade glutaraldehyde in 0.1 M sodium cacodylate buffer (pH 7.3) for 10 min to fix the bacteria on the surface.

To remove water slowly, samples were dehydrated for 10 min in ethanol solutions with increasing concentrations of 20, 30, 40, 50, 60, 70, 90, 95, and 100%. After dehydration, hexamethyldisilizane (HMDS; Sigma-Aldrich) was used to remove the alcohol.

Samples were then coated with Au before SEM imaging by a Zeiss MERLIN field-emission SEM instrument (Carl Zeiss GmbH, Oberkochen, Germany). All samples were scanned at a voltage of 10 kV and a working distance of 9 mm.

#### 2.5.3 Confocal Laser Scanning Microscopy (CLSM)

CLSM was applied to characterize the live or dead bacteria on the various coated surfaces. Briefly, after 24 h of bacteria culture (20 μL, 10^5^ bacteria/mL) with each substrate (described in section 2.5.1), surfaces were gently washed with PBS to remove nonadherent bacteria. Samples were then stained with the live/dead BacLight bacterial viability kit (Invitrogen) according to the manufacturer’s instructions. The kit included a solution of SYTO 9, the green-fluorescent nucleic acid stain, and propidium iodide, the red-fluorescent nucleic acid stain, in dimethyl sulfoxide (DMSO). Equal volumes of the components were mixed thoroughly in a microfuge tube. A total of 5 μL of the dye mixture was added to each sample and incubated in the dark at room temperature for 15 min. The excitation/emission maxima of the dyes are about 480/500 nm for the SYTO 9 stain and 490/635 nm for propidium iodide. The samples were characterized by noninverted CLSM using a Leica TCS SP8 microscope. The viable bacteria appeared fluorescent green, while the nonviable bacteria appeared fluorescent red.

### 2.6 Cell Viability and Compatibility Assessment

#### 2.6.1 Cell Culture

The human fetal osteoblast cell line hFOB 1.19, obtained from ATCC, was cultured in a 1:1 mixture of Ham’s F12 sedium and Dulbecco’s modified Eagle’s medium (DMEM) without phenol red, supplemented with 2.5 mM l-glutamine, 15 mM 4-(2-hydroxyethyl)-1-piperazineethanesulfonic acid, and 0.3 mg/mL G418. This medium was further supplemented with 1% penicillin–streptomycin and 10% fetal bovine serum. Cells were incubated at 34 °C, and upon reaching 90% confluence, the cells were rinsed with sterile PBS and treated with a 0.25% (w/v) Trypsin–0.53 mM ethylenediaminetetraacetic acid (EDTA) solution for 10 min at 34 °C to detach them from the tissue culture surface. Cells were then transferred to a centrifuge tube containing the culture medium and centrifuged at approximately 125*g* for 5 min. The cell pellets were resuspended and subcultured at a ratio of 1:4. Cells from passage 3 were utilized in all experiments. The culture medium, supplements, and all reagents for cell culture were purchased from ThermoFisher Scientific Inc.

#### 2.6.2 Quantification of the Cell Viability Using MTT Assay

The cytotoxicity of coated titanium surfaces was assessed using methylthiazolyldiphenyltetrazolium bromide (MTT) assay supplied by Sigma-Aldrich. The cell viability on each titanium sample was measured at 1, 3, 5, and 7 days postseeding. Cells were cultured as described above, and titanium samples were washed twice with PBS and briefly soaked in ethanol before being transferred to new 24-well plates. Cells were then seeded onto the coated disks (10 mm diameter), previously positioned in 24-well plates, at a density of 20000 cells/disk, using a 20 μL drop. Following a 30 min incubation at 34 °C to facilitate cell attachment, an additional medium was added to reach a total volume of 500 μL for further culture. The culture medium was refreshed every 48 h.

A blank growth medium served as the background control. At specific intervals during the incubation period at 34 °C, the medium in each well was substituted with 100 μL of MTT solution (0.5 mg/mL in supplemented DMEM) and incubated for 4 h. Subsequently, the solution was aspirated, and the insoluble formazan crystals were dissolved in 100 μL of DMSO. After a 15 min incubation period, the absorbance was measured at a wavelength of 570 nm using an ELISA microplate reader and expressed as a percentage relative to the absorbance of the nontoxic control. Due to the light sensitivity of the MTT reagent, all procedures were conducted in the dark.

#### 2.6.3 Cell Morphology

Following a similar cell culture procedure as described above, the prepared samples were cultured with hFOB (20000 cells/cm^2^) to observe the cell morphology after 3 and 7 days. After culturing, surfaces were gently washed with PBS and then fixed with 4% paraformaldehyde in PBS (ThermoFisher Scientific) for 40 min. Following fixation, samples were sequentially dehydrated with ethanol solutions [30, 50, 70, 80, 90, and 100% (v/v)] for 10 min each. HMDS was used to remove the alcohol. Finally, the surfaces were sputter-coated with Au, and the morphology of the cells was examined by using a Zeiss MERLIN field-emission SEM instrument (Carl Zeiss GmbH).

### 2.7 Statistical Analyses

The results are presented as mean ± standard deviation (SD) from three independent repeats, with *n* ≥ 3 indicated. Statistical analysis was performed using SPSS with a one-way analysis of variance (ANOVA) with Tukey’s posthoc method to evaluate significant differences between groups. A *p* value of <0.05 was considered statistically significant (*p < 0.05, **p < 0.01, and ***p < 0.001).

## 3 Results and Discussion

This study aimed to develop a biomimetic enzyme-responsive antimicrobial coating by exploiting the elastolytic degradation potential of the recombinant HELP biopolymer. A stepwise procedure was employed to initially functionalize a silanized titanium surface with HELP biopolymer, followed by the immobilization of KR-12 on the HELP linker. Various strategies for the covalent bonding of biological molecules to titanium surfaces have been documented, with silane chemistry particularly prominent.^48^ Among these approaches, APTES stands out due to its bifunctional nature. APTES possesses three alkoxy groups capable of forming siloxane bonds with titanium hydroxyl groups, while its nucleophilic amine groups serve as anchoring sites for the further attachment of bioactive compounds via cross-linking methods.^49^ However, note that the siloxane bond in an APTES coating exhibits hydrolytic instability in aqueous environments, resulting in a gradual loss of covalently attached silane molecules over time. Moreover, the silanization process yields a multimolecular layer, which may impact the alignment of peptides and introduces challenges in predicting their orientation.^50^

Following silanization, the carboxyl groups of HELP reacted with primary amine groups of APTES on the surface via an EDC/NHS activating process. As a result, the amine groups of HELP were primed as anchoring points for subsequent AMP immobilization. In this study, various ratios of HELP and peptide were explored, and the reported concentrations represent the optimized and finalized parameters, which have been utilized for subsequent characterizations.

### 3.1 Surface Characterization by SEM, FTIR, AFM, XPS, and WCA

**Figure 2:**
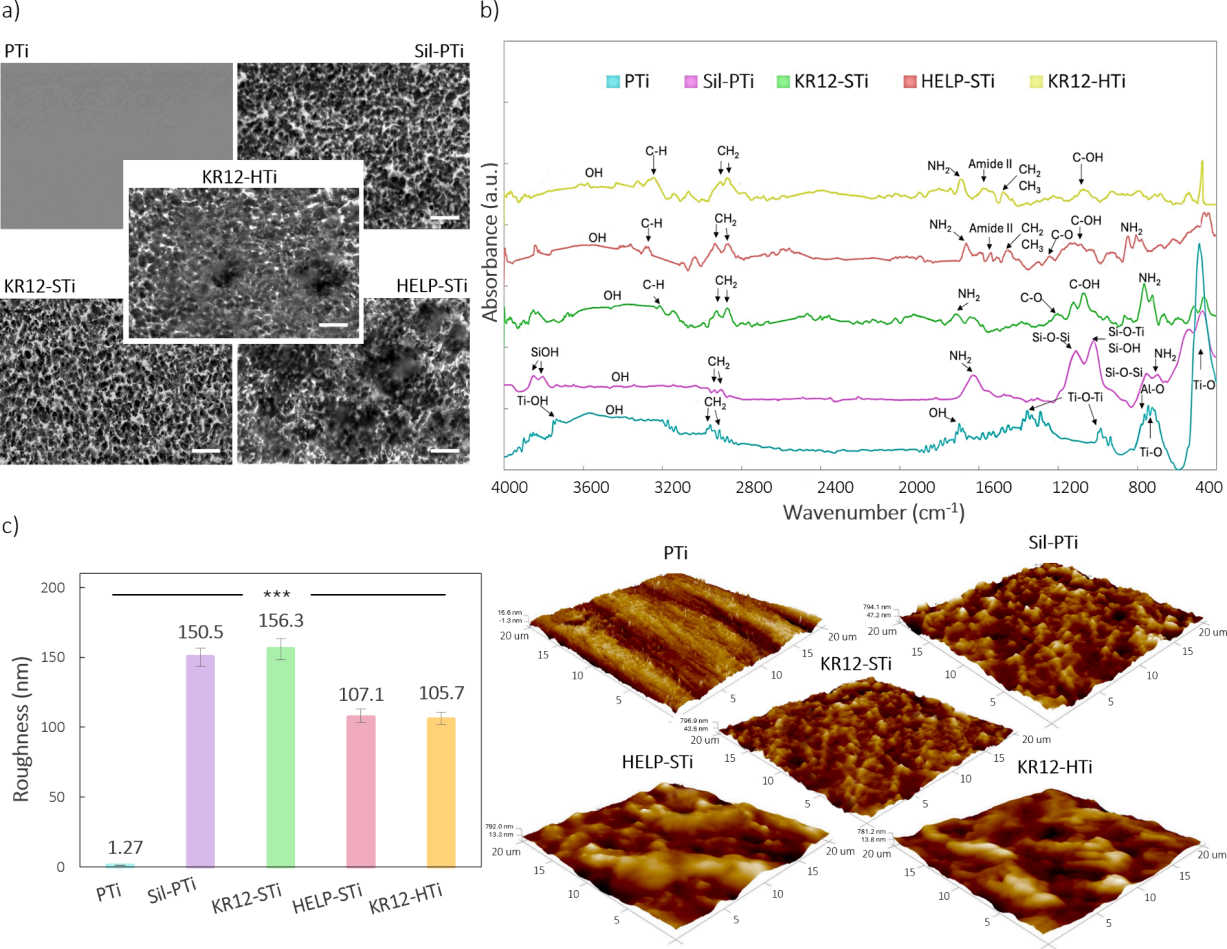
Surface characterization of coated titanium surfaces. (a) SEM micrographs depicting the surface morphology, with a scale bar of 10 μm, (b) ATR-FTIR spectra, and (c) mean roughness (*R*_a_) and morphology analyzed by AFM 3D imaging. *** denotes the statistical significance (*p* < 0.001) between the polished titanium (PTi) and all modified surfaces.

The morphological characteristics of the surfaces were examined using SEM subsequent to each phase of surface functionalization, as depicted in Figure 2. Initially, untreated polished titanium (PTi) specimens displayed a smooth surface, albeit they exhibited scarcely detectable scratches attributed to experimental manipulation (Figure 2a). Following the silanization initialized by hydroxylation through etching, a remarkable alteration in surface topography was observed. Sil-PTi specimens subjected to the silanization process exhibited a roughened surface morphology characterized by a distinct ridge-like microstructure.

Direct covalent immobilization of the KR-12 peptide (KR12-STi samples) elicited no observable impact on the surface morphology compared to Sil-PTi. In contrast, HELP attachment on the surface (HELP-STi) revealed the deposition of an opaque polymeric layer discernible on the rough titanium surface (Figure 2a). Furthermore, upon the introduction of KR-12 peptides after HELP attachment, KR12-HTi samples showed no apparent changes, except for the microstructured titanium surface becoming nearly entirely covered by a thin, blurred layer. This occurrence can be ascribed to the disentanglement of the HELP biopolymer during the peptide immobilization process.

Utilizing ATR-FTIR spectroscopy provided an essential understanding of the molecular composition and structural changes throughout the coating preparation process. The obtained IR spectrum exhibited vibration bands characteristic of different stages of the coating. The distinctive bonds associated with each peak are marked in the spectra, as shown in Figure 2b. For PTi, a prominent peak at 488 cm^–1^ is attributed to the Ti–O stretching band, characteristic of Ti6Al4 V, along with bands at 735 and 786 cm^–1^ corresponding to the Ti–O and Al–O bonds.^51,52^ Peaks at 998 and 1413 cm^–1^ are attributed to the Ti–O–Ti stretching vibration bands.^19,53^ A broad vibration band between 3150 and 3680 cm^–1^ arises from the stretching vibrations of the O–H groups of physisorbed water molecules on the polished surface, while a weaker band at 1694 cm^–1^ is associated with the bending vibration of the corresponding free −OH groups. Peaks at 2905 and 2943 cm^–1^, falling between 3000 and 2800 cm^–1^, indicate C–H stretching vibration absorbance bands, possibly due to equipment contamination and air adsorption.^53,54^

Following silanization with APTES, the IR spectra still showed broad bands corresponding to O–H bending, albeit with decreased intensity, indicating interaction between TiO_2_ and APTES molecules. The silanized surface exhibited a band at 1612 cm^–1^ corresponding to the deforming vibrations of primary amino groups and a band at 998 cm^–1^ specific to stretching vibrations in the Si–O bond of silanol groups (Si–OH) and Si–O–Ti, demonstrating APTES immobilization through hydroxyl groups.^55^ Additionally, the presence of a band at 1136 cm^–1^, specific to Si–O–Si, suggests binding between multiple APTES molecules via ether bridges, enhancing the stability of the immobilization process.^56^ Sharp bands at 3797 and 3822 cm^–1^ indicate free silanol groups from organosilane compounds on the sample surface.^55^ Furthermore, symmetric and asymmetric stretching vibrations characteristic of methylene groups from APTES are observed at 2911 and 2844 cm^–1^, respectively.^57^

After HELP and KR-12 immobilization, the broadness of the peak makes it challenging to distinguish individual peaks. However, characteristic peaks affirm the effectiveness of the grafting process. Sharp peaks at 2911 and 2948 cm^–1^ represent CH and CH_2_ stretching and bending vibrations, while larger peaks between 1548 and 1620 cm^–1^ correspond to amide II.^54,55^ Moreover, stretching vibrations of C–H bonds are detected at 3231, 3289, and 3273 cm^–1^ in the KR12-STi, HELP-STi, and KR12-HTi samples, respectively. A peak at 1489 cm^–1^ coincides with CH_2_ and CH_3_ bending vibrations in the backbone of the grafted HELP biopolymer. Additional strong peaks at 1309 and 1261 cm^–1^ correspond to the C–O stretch, suggesting the presence of ester and acid.^58,59^ The primary amine group exhibited a doublet at 804 and 747 cm^–1^ for KR12-STi and 883 and 811 cm^–1^ for HELP-STi, indicating deformation vibration, while the C–O bond showed stretching vibrations at 1083 and 1018 cm^–1^.^54,60^ The broad absorption between 3650 and 3050 cm^–1^, representing the −OH stretching region, cannot solely be attributed to −OH peaks from the carboxylic acid groups of peptides and HELP biopolymer but also to the presence of physisorbed water molecules.

Surface properties, including morphology and roughness, are critical factors affecting cell behavior, such as adhesion, proliferation, and differentiation. AFM was employed to precisely evaluate these surface characteristics due to its ability to provide high-resolution topographical information. The surface topography was assessed after each step of the surface coating. Before the reaction, the titanium substrates exhibited a smooth morphology with very low roughness (1.2 ± 0.2 nm) due to mirror-finish polishing (Figure 2c). After silanization, the Sil-PTi sample displayed notable alterations in surface morphology and roughness, consistent with the SEM micrographs showing a similar microstructure pattern for Sil-PTi (Figure 2a). The silanized surface exhibited a statistically significant increase in roughness (150.5 ± 8.1 nm) due to the surface pretreatment with the etching solution. Conversely, the immobilization of KR-12 onto the silanized surface, denoted as the KR12-STi sample, exhibited negligible alterations in both morphology and roughness. This observation suggests that the influence of the short KR-12 molecules on these surface characteristics was minimal.

HELP integration resulted in an evident ridge-like structure (Figure 2c), demonstrating the successful assembly of HELP biopolymer onto the titanium surface. HELP-attached surfaces exhibited reduced *R*_a_ values of 107.1 ± 11.4 nm compared to those of Sil-PTi. This reduction suggests that the high-molecular-weight biopolymer of HELP serves as a covering layer for surface pores, effectively concealing sharp and large edges and imparting a smoother appearance. Following the KR-12 peptide integration for KR12-HTi, the surface morphology did not change significantly, with bulk morphology still observable. The minimal change in the morphology and roughness observed compared to the previous step is likely due to the superior coverage provided by HELP, attributed to its larger molecular size (about 30-fold). This resulted in visible aggregation of HELP on all silanized surfaces, while the impact of the shorter molecules of KR-12 was negligible. By immobilizing HELP and KR-12 on titanium surfaces and subsequently examining them with AFM, we were able to assess how these modifications influenced the surface properties and, by extension, the near-surface environment for cells. This allowed us to determine the suitability of the modified surfaces for promoting cell adhesion and proliferation.

**Figure 3:**
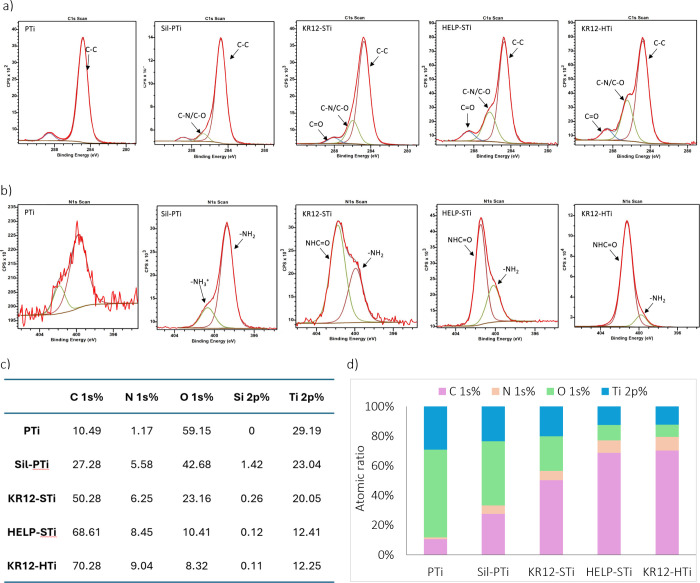
XPS analysis results for characterization of the surface elemental composition after the modification steps during silanization, HELP attachment, and KR-12 immobilization: (a) high-resolution C1 s spectra and (b) N 1s spectra of PTi, Sil-PTi, KR12-STi, HELP-STi, and KR12-HTi, (c) relative atomic ratios of chemical compositions, and (d) a graphic illustration of different element ratios for all samples.

XPS measurements were conducted to validate the successful functionalization and grafting at each stage of sample coating by assessing the chemical groups present on the surface. These measurements aimed to determine the quantity of primary amine groups present on the silanized surface, serving as attachment points for the subsequent immobilization steps. The XPS survey spectra (Figure S1) for each modification stage exhibited characteristic peaks corresponding to carbon (C), oxygen (O), nitrogen (N), silicon (Si), and titanium (Ti). Additionally, minimal traces of aluminum, vanadium, sulfur, and phosphorus were detected, likely originating from the Ti6Al4 V alloy and contaminants introduced during sample preparation. Higher-resolution scans were conducted to further investigate the chemical bonding on the surfaces, as presented in Figure 3.

In the XPS region of the PTi surface, the titanium (Ti 2p) peak is typically identified at around 458.7 eV. The oxygen (O 1s) peak predominantly originates from the titanium oxide layer on the surface. Additionally, the C 1s scan reveals a sharp peak attributed to residues from the polishing step as well as the presence of carbon contamination from the equipment chamber, which is unavoidable in XPS measurements. This peak indicates the presence of C–C or C–H bonds, constituting approximately 10.49% of the atomic ratio on the surface.

Following the silanization of titanium surfaces, the XPS survey in Figure S1 revealed noticeable Si 2s and Si 2p signals in Sil-PTi compared to PTi, indicating the successful grafting of APTES onto the titanium surface. The elemental composition analysis of silanized titanium samples showed an increase in N% and C% to 5.58% and 27.28%, respectively, and a decrease in O% to 42.08%. The high-resolution C 1s spectrum of the silanized surface exhibited a primary peak at 285 eV attributed to C–C and C–H bonds, originating from the presence of the propyl chain. Moreover, the high-resolution N 1s spectrum displayed two main peaks attributed to protonated and nonprotonated amine groups in APTES. By measurement of the percentage of N 1s in Sil-PTi (5.58%) and the ratio of NH_2_ to the overall N on the surface (83.6%) after deconvolution of the N 1s scan, the calculated percentage of primary amine groups on the surface, which act as anchoring points for further steps, is 4.67%.

Upon covalent immobilization of the KR-12 peptide onto the silanized titanium surface, the amine groups on the surface formed covalent bonds with the carboxyl group of the KR-12 peptide. This led to a notable increase in the C 1s and N 1s signals, accompanied by a decrease in the O and Ti signals (Figure S1). Additionally, the signals corresponding to Si 1s and Si 2p became nearly absent. Deconvolutions of the C 1s and N 1s peaks from high-resolution XPS spectra are presented in parts a and b of Figure 3, respectively. The C 1s signal of KR12-STi exhibited three peaks: one at a binding energy of 284.9 eV associated with C–C bonds, another at 286.2 eV related to C–O/C–N bonds, and a third at 288.3 eV for C=O bonds. The XPS N 1s scan of KR12-STi revealed two peaks: one at 399.8 eV corresponding to amine bonds (−NH_2_) of side chains and one at 401.6 eV arising from amide bonds (−NHCO), confirming the covalent immobilization of KR-12 on the surface.^19^ Consequently, the N signal from the amide bonds, which are more abundant than those from the amines, appears to be more intense, as anticipated.

A subsequent increase in the N content, totaling 6.25 and 8.45%, was noted for KR12-STi and HELP-STi, respectively, attributed to the presence of amine and amide groups within the KR-12 and HELP molecular chains. Quantitatively, the N/Ti atomic ratio rose from 0.24 to 0.31 following the immobilization of the KR-12 peptide on the silanized surface and from 0.68 to 0.73 for HELP-STi and KR12-HTi. Overall, higher N/Ti and C/Ti atomic ratios were evident for HELP-STi compared to KR12-STi surfaces, as anticipated due to the larger molecular weight of the HELP polymers relative to that of the AMP KR-12. The alterations in the atomic ratios of all elements are presented in Figure 3c,d. The C 1s and N 1s scans of KR12-HTi and KR12-STi have similar peaks at identical binding energies, with variations in percentages, indicating no significant differences.

The FTIR and XPS findings illustrate the successful chemical grafting of KR-12 and HELP onto the silanized surfaces.

**Figure 4:**
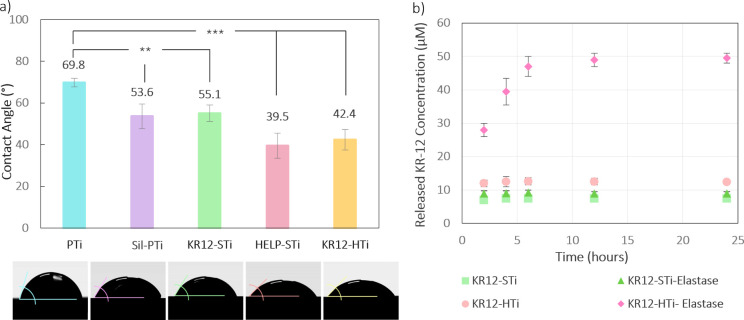
Quantitative measurement of (a) WCA of each titanium substrate (**p < 0.01 and ***p < 0.001) and representative images of the water drops on the different coatings and (b) KR-12 release from coatings in the absence and presence of elastase. Reactions were carried out at 37 °C for 24 h. Following incubation, the proteolytic activities were detected by absorbance readings at 585 nm. The results were the mean ± SD of three independent experiments performed in triplicate.

The hydrophilicity of each substrate was assessed through the WCA analysis presented in Figure 4. The titanium substrate exhibited a contact angle of 69.8 ± 4.7°. Subsequent silanization resulted in a decreased contact angle for Sil-PTi, measuring 53.6 ± 8.3°, indicating increased hydrophilicity compared to the PTi substrate (Figure 4). Despite the expectation of increased WCA due to the presence of the propyl chain in APTES, the hydroxyl groups and rough surface morphology led to a final reduction in WCA and a consequent increase in hydrophilicity. Covalent immobilization of the KR-12 peptide on the silanized titanium surface slightly decreased the hydrophilicity (55.1 ± 6.2°). This reduction can be attributed to the hydrophobic amino acids present in the KR-12 peptide, such as leucine, isoleucine, and valine. Moreover, when the peptide is tethered via its C-terminal, the lysine in KR-12 becomes exposed on the surface. Lysine is a positively charged amino acid, housing a primary amine group. Although the amine group has the potential to engage in hydrogen bonding with water, it typically exhibits lower hydrophilicity in comparison to other polar functional groups such as hydroxyl (−OH) or carboxyl (−COOH). Consequently, this feature may lead to a slight increase in the overall hydrophobicity of the immobilized peptide. Nonetheless, KR12-STi can still be categorized as a hydrophilic surface due to its low WCA. It should be noted that there is no straightforward explanation relating the properties of the AMP or its orientation to the resulting WCA of the grafted surface.

The integration of HELP linkers notably increased the hydrophilicity of the titanium surface. According to previous observations,^61^ the amphipathic nature of elastin-like domains enables them to reconfigure at the solid/water interface in response to the interactions with water molecules. This explains the significant reduction in the WCA observed for HELP-STi surfaces (39.5 ± 7.7°). The negligible difference in the hydrophilicity between HELP-STi and KR12-HTi (42.4 ± 6.9°), where KR-12 is absent and present, respectively, suggests that the interaction with water is mainly driven by the exposure of the HELP biopolymer. This is likely due to the longer chains of HELP, which results in a higher surface coverage compared to that of KR-12. As indicated, incorporating the HELP linker enhances the surface hydrophilicity, potentially promoting interaction with the biological system.

### 3.2 Calculation of the Peptide Density

Due to the molecular structure resemblance between KR-12 and HELP, both composed of amino acids, precise differentiation and quantification of the immobilized peptide amount utilizing XPS and FTIR pose challenges. Hence, an alternative technique using the Fmoc/t-Bu strategy has been employed to quantify the immobilized peptide content, as described in Experimental Methods section 2.3.

Utilization of the Fmoc/t-Bu strategy has become prevalent due to the advantageous properties of the Fmoc moiety, which exhibits absorption in the UV region. Figure S2 illustrates the removal of the Fmoc group via a β-elimination reaction induced by treatment with a secondary amine, piperidine. This reaction yields the highly reactive DBF, which subsequently reacts with excess piperidine to form the DBF adduct, characterized by a distinctive UV absorbance peak at 301 nm. A calibration curve relating the concentration of the DBF-based adduct to absorbance was established by measuring UV absorption across a range of concentrations (10–310 μM). This curve exhibited a linear relationship with a molar absorption coefficient of 8.141 L/mol·cm at λ = 301 nm. Both the calibration curve and the reaction scheme are provided in Figure S2. The absorbance of each solution (*n* = 3) was assessed at λ = 301 nm and correlated with the established calibration curve. The results revealed that the quantities of Fmoc groups present on surfaces, in the absence or presence of the HELP linker, were 61.34 and 256.52 nmol, respectively, with reaction yields of 11% and 46%. KR12-HTi displayed a 3-fold rise in KR-12 loading compared to KR12-STi. This increase can be attributed to the higher concentration of primary amine groups in the side chains of the HELP linker acting as anchors.

### 3.3 KR-12 Release Triggered by Elastase

This work aimed to explore the potential of HELP-based coatings as a delivery system capable of releasing the antimicrobial agent under an enzymatic stimulus from the environment. As shown in Figure 4, a high release of KR-12 from KR12-HTi substrates was detected after incubation with elastase for 6 h, corresponding with the high elastolytic degradation potential of HELP. It is well-known that this enzyme efficiently degrades stretches of alanines,^28^ like those presented in the hydrophilic domains of HELP.

As expected, elastase exhibited very high elastolytic activity because it was able to degrade the HELP linkers on the KR12-HTi samples, detected by higher peptide release from the substrates than samples without added enzyme. KR12-STi in the presence and absence of elastase, under similar conditions, showed a slight release of 7–13.1 μM in 24 h, suggesting that elastase did not have an impact on KR-12.

After 24 h, 71.2 μM peptides were released from the KR12-HTi sample. This amount is lower than the overall amount of immobilized peptides, suggesting that the grafted HELP macromolecules are less sensitive to the proteolytic activity compared with the free form of HELP in solution. In addition, a more pronounced increase in peptide release could be expected at a higher elastase concentration. Our results suggest that a substantial part of the immobilized Fmoc-labeled KR-12 was effectively released under the proteolytic stimulus.

### 3.4 Antimicrobial Activity Assay

**Figure 5:**
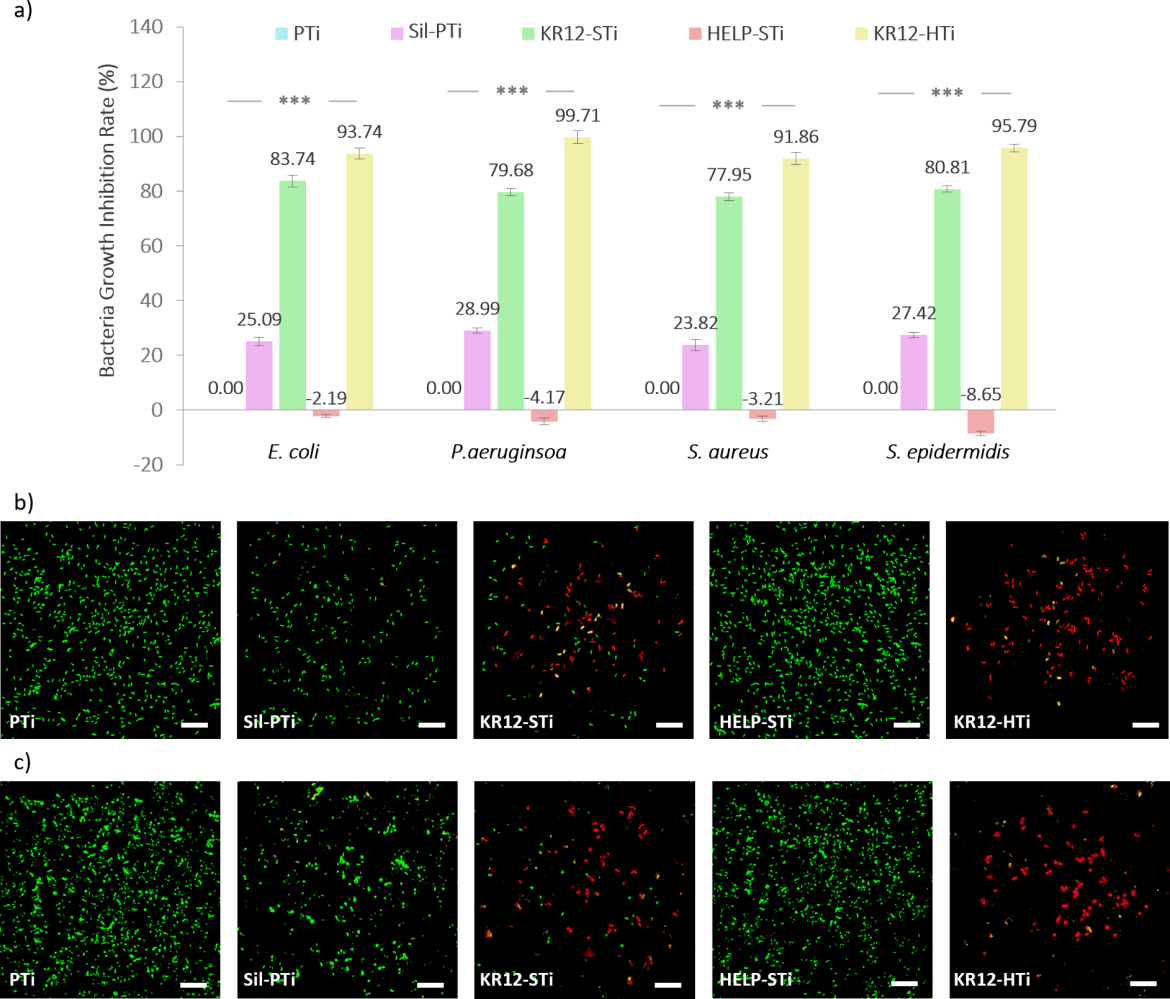
Assessment of the antimicrobial activity: (a) bacterial growth inhibition ratio of the indicated surface coatings against *E. coli*, *P. aeruginosa*, *S. aureus*, and *S. epidermidis*. PTi samples were considered as controls showing 0.00% inhibition ratio. * denotes *p* < 0.05, ** denotes *p* < 0.01, *** denotes *p* < 0.001 (error bars represent the SD from three independent experiments, each performed in triplicate). Representative fluorescence live/dead assay images of (b) *E. coli* and (c) *S. aureus* cultured on different coatings (scale bars = 6 μm; green bacteria, live; red bacteria, dead).

*S. aureus* and *S. epidermidis*, as Gram-positive bacteria, and *E. coli* and *P. aeruginosa*, as Gram-negative bacteria, were specifically chosen for this study to assess the antimicrobial effectiveness of AMP-immobilized surfaces for orthopedic applications. The antibacterial activity of the substrates was evaluated through a DCT, where 10^5^ CFU/mL of each bacterial strain was added onto the surfaces and then incubated for 24 h. The antimicrobial efficacy of the substrates was quantified and expressed as a percentage of bacterial killing compared to a reference (PTi) after 24 h. The bacterial growth inhibition ratio was calculated by subtracting the colony numbers of each sample from the colony numbers of PTi and then dividing by the colony numbers of PTi, as reported in Figure 5a.

**Figure 6:**
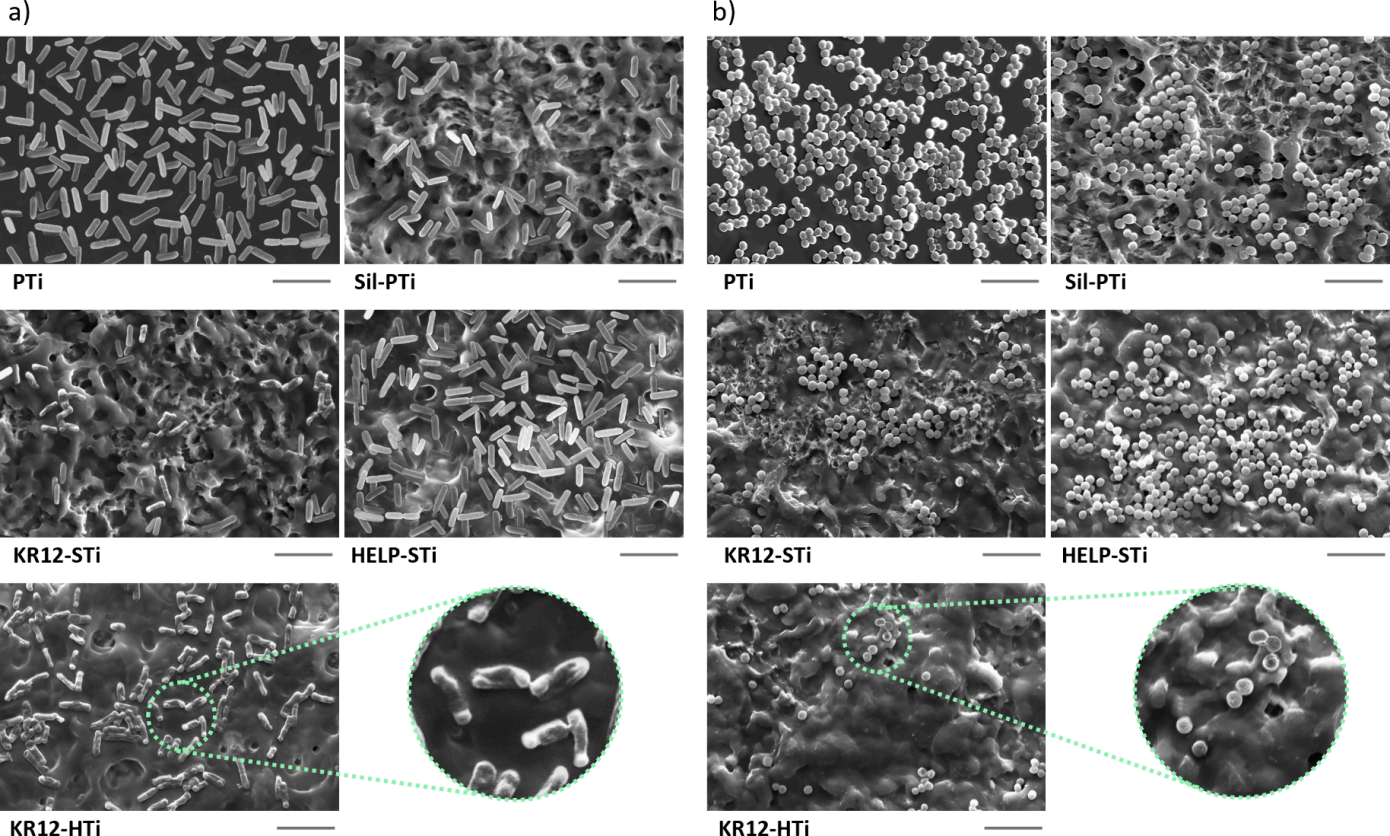
SEM characterization of the bacterial growth of substrates. SEM images of (a) *E. coli* and (b) *S. aureus* on indicated substrates. All results were taken after 24 h of incubation with the surface. Scale bars = 5 μm. AMPs attached to KR12-HTi and KR12-STi showed adequate bactericidal activity to disrupt the membranes of both *E. coli* and *S. aureus* bacteria.

PTi exhibited no antibacterial activity, while Sil-PTi showed mild antibacterial effects, with inhibition ratios ranging from 25% to 29% across all bacterial strains. This modest growth inhibition could be attributed to the rough morphology of the silanized surface, which possesses sharp edges that hinder bacterial attachment. This hypothesis was further supported by live/dead staining assays and SEM micrographs (Figures 5b,c and 6). Following the modification of Sil-PTi with KR-12, KR12-STi exhibited substantial antibacterial effects, with inhibition ratios of 83.74%, 79.68%, 77.95%, and 90.81% against *E. coli*, *P. aeruginosa*, *S. aureus*, and *S. epidermidis*, respectively. Overall, the introduction of KR-12 significantly endowed titanium surfaces with antibacterial efficacy against Gram-positive and Gram-negative bacteria. This is consistent with its demonstrated killing efficiency against Gram-negative bacteria in solution, reported in previous findings.^38,62^

KR12-HTi demonstrated the highest antibacterial activity, with inhibition ratios of 93.74%, 99.71%, 91.86%, and 95.79% against *E. coli*, *P. aeruginosa*, *S. aureus*, and *S. epidermidis*, respectively (Figure 5a). The proposed mechanism suggests that the antibacterial action of AMPs involves the insertion of positively charged peptides into bacterial cell membranes, leading to the disruption and eventual death of bacteria. AMPs with HELP biopolymers as linkers are hypothesized to interact with bacterial cell membranes more easily, thereby exhibiting superior antibacterial properties, as supported by previous studies using other polymer linkers.^63,64^ Compared to KR12-STi, KR12-HTi, exhibiting flexible HELP linkers, showed increased antimicrobial activity by an average of 14.6% across all bacterial strains. This is attributed to a higher AMP density as well as an enhanced mobility for effective bacterial interaction.

AMPs primarily exert antibacterial effects through electrostatic interference and destabilization of bacterial membranes. In the absence of a linker, KR12-STi destabilized bacterial membranes, increasing their susceptibility to physical puncture. It has been reported that the rough surfaces, similar to those induced by the silanization process, could play a crucial role in physically puncturing destabilized bacteria, particularly effective against Gram-negative bacteria.^65^ Compared to Gram-positive bacteria, Gram-negative bacteria are more susceptible to surface interactions and membrane disruption, due to a thinner peptidoglycan layer and possession of both inner and outer membranes.^65^ KR12-HTi demonstrated superior bactericidal activity against *P. aeruginosa* (inhibition ratio of 99.71%), which is notably higher than that observed for other strains. This higher inhibition could be attributed to the elastase production capacity of *P. aeruginosa*,^66^ stimulating AMP release.

To assess various substrates’ bactericidal efficacy and adherence properties, we employed a live/dead staining assay, as depicted in Figure 5b,c. In the live/dead assay, dead bacteria emitted red fluorescence, while live bacteria emitted green fluorescence. *S. aureus* and *E. coli* were selected as model bacteria of Gram-positive and Gram-negative bacteria to present the concept of bacterial behavior on different substrates. Consistent with the DCT results, live and dead staining revealed that PTi exhibited no antimicrobial activity against *S. aureus* and *E. coli*, with all bacterial colonies remaining viable and stained green.

Live/dead assays of Sil-PTi indicated that the attached bacteria were alive and green (Figure 5b,c). However, the number of attached bacteria was reduced with respect to the control sample, PTi, possibly due to the surface roughness, resulting in mild growth inhibition. Despite their rough morphology, both HELP-STi and KR12-HTi surfaces demonstrated greater bacterial attraction compared to Sil-PTi. The presence of the HELP moiety seemed to attract microorganisms. However, most of the adhered bacteria on KR12-HTi were dead (Figure 5b,c).

Both HELP-STi and KR12-HTi surfaces exhibited bacterial affinity higher than that of Sil-PTi. The inclusion of HELP as a linker appeared to increase the level of bacterial adhesion. However, a significant proportion of bacteria that adhered to KR12-HTi were found to be dead. In addition, we conducted SEM analyses to characterize the morphology of *E. coli* and *S. aureus* after 24 h of culture on the surfaces, as represented in Figure 6. PTi and Sil-PTi surfaces exhibited typical shapes with intact cell walls, suggesting a lack of antimicrobial activity. Despite the absence of biofilm formation on PTi, many bacteria adhered to and aggregated on the surface. Sil-PTi, however, slightly inhibited bacterial colonization compared to PTi, consistent with the DCT and live/dead staining results.

A cell morphology similar to that of Sil-PTi was observed for HELP-STi, coupled with increased bacterial attachment, suggesting HELP’s supportive role in facilitating bacterial adhesion without detrimental effects. SEM images revealed that, compared to PTi and Sil-PTi, *S. aureus* cultured on KR12-STi and KR12-HTi showed distinct morphological changes characterized by shrunken and disrupted membranes, indicative of damage. Similarly, *E. coli* on KR12-HTi and KR12-STi exhibited corrugated, distorted, and lysed morphology, suggesting membrane damage possibly induced by the AMP. It can be assumed that AMPs immobilized on KR12-HTi provided sufficient mobility to permeate the bacterial membrane, leading to bactericidal activity. On the other hand, the positively charged surfaces of KR12-STi facilitated electrostatic interactions with bacteria, thereby enhancing the efficacy of AMP action.

While SEM data alone cannot ascertain the precise mechanism of KR-12 action on cellular membranes, they strongly suggest that significant damage can be inflicted by the immobilized KR-12. Future studies employing transmission electron microscopy will provide further insights.

### 3.5 Cytocompatibility and Cell Adhesion

The cytocompatibility of the modified titanium surfaces was evaluated using hFOB 1.19 cells, an osteoblast cell line of human origin, frequently employed in studies of osteoporosis, bone tissue engineering, and orthopedic biomaterials. The cell viability was assessed via MTT assay, which indirectly measures cell proliferation by assessing the metabolic activity. It also provides a measure of cytocompatibility by determining whether cells remain metabolically active when exposed to materials. Previous studies have investigated the cytocompatibility of KR-12 peptides in solution, revealing no cytotoxic effects within a specific concentration range.^15,38,62^ However, at higher AMP concentrations, the cell viability decreases. It is crucial to find a balance in loading antibacterial agents to optimize efficiency while mitigating potential cytotoxicity. Although a high loading density of antibacterial agents can effectively eradicate bacteria, they also pose the risk of being cytotoxic.

In this study, the cell viability relative to controls was measured at three time points (3, 5, and 7 days). MTT assay results indicated higher proliferation rates of hFOB cells on titanium disks with different coatings compared to the control (standard TCP) across all time points. This aligns with previous findings on the cytocompatibility of HELP and the low toxicity of KR-12 peptide.^15,29^ Despite exhibiting potent antimicrobial activity, KR12-HTi showed biocompatibility comparable to that of PTi and higher than that of KR12-STi surfaces. Sil-PTi and KR12-STi samples also demonstrated support of cell growth, indicating strong cell attachment despite minimal bacterial adherence attributed to the rough morphology of the surfaces. Osteoblasts, being larger and more resilient than bacteria, were less affected by surface roughness, thereby indicating the inherent cytocompatibility of silanized surfaces.

After 3–7 days of incubation, the cell viability was significantly higher on HELP-STi and KR12-HTi compared to PTi, indicating good cytocompatibility even in the presence of grafted KR-12. This excellent cell-adhesion-promoting activity, attributed to HELP, aligns with the results of recent studies on its biological interactions at the molecular level.^67^ These studies specifically worked on the fusion of HELP with AMPs to enhance cellular attachment by facilitating integrin binding and interactions with ECM components. However, slightly lower viability was observed on KR12-HTi compared to HELP-STi, possibly due to the slight decrease of hydrophilicity observed in KR12-HTi, as discussed earlier in section 3.1. In contrast to KR12-HTi, statistically significant variations in the metabolic activity were detected between KR12-STi and PTi at all time points.

**Figure 7:**
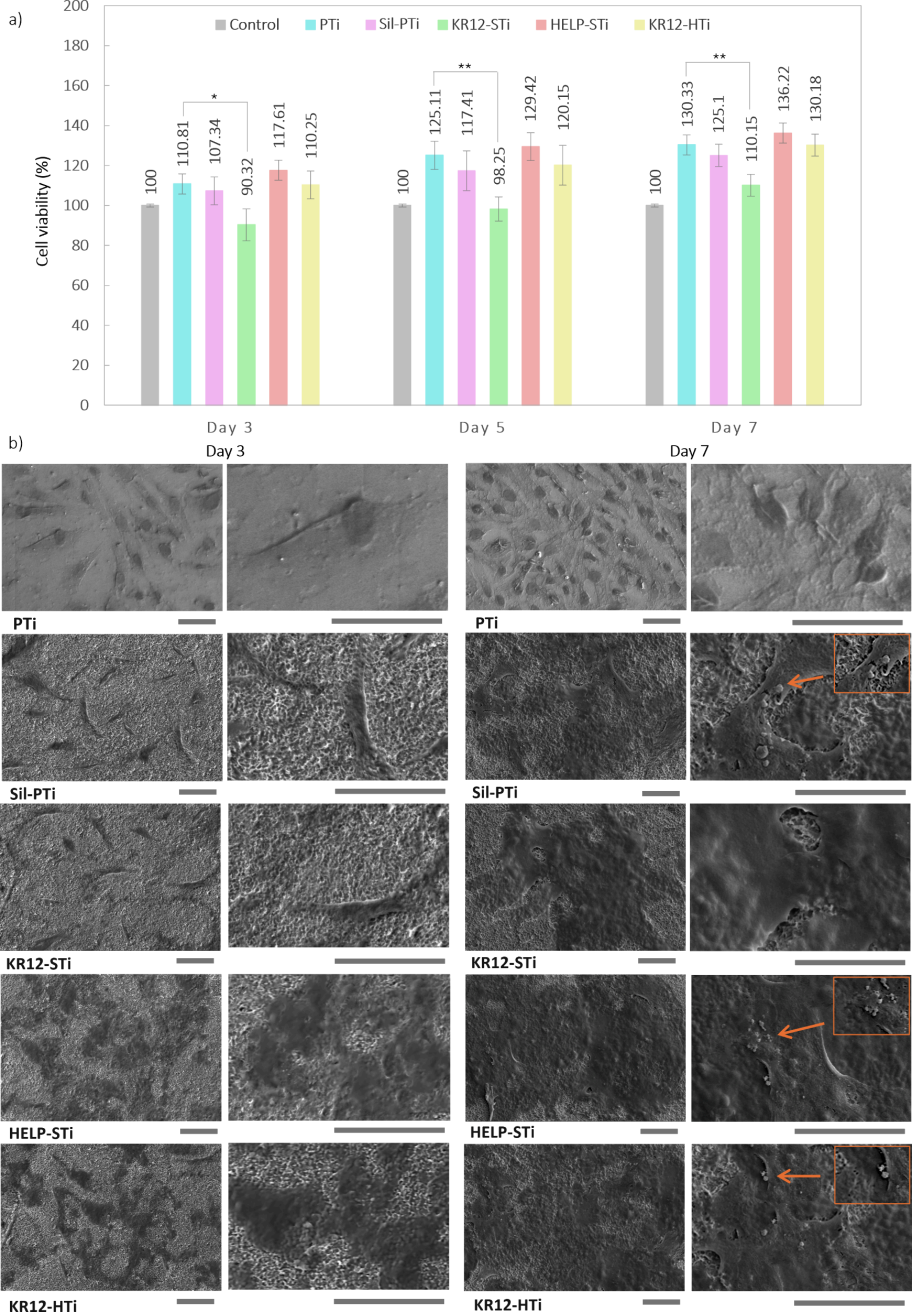
Cytocompatibility and cell morphology study: (a) Cell viability results of the MTT test of hFOB cells cultured on different substrates after 3, 5, and 7 days of culturing, * denotes significant differences (*p* < 0.05) compared with PTi. (b) SEM micrographs showing the morphology of the seeded hFOB cells on days 3 and 7. Scale bars = 20 μm. By day 7, the cells exhibited a spread morphology with filopodia extending toward adjacent cells, accompanied by the formation of mineralized deposits (depicted by arrows) on KR12-STi, HELP-STi, and KR12-HTi surfaces.

Cell adhesion and the interaction between cells and coatings play a pivotal role in successfully integrating implants into native bone tissue.^68,69^ The evaluation of cell distribution and adhesion on each surface after 3 and 7 days of incubation was performed using SEM. The SEM images in Figure 7, captured on days 3 and 7, depict normal attachment and growth of hFOB cells on untreated PTi surfaces. Compared to PTi, the extent of cell spreading on Sil-PTi and KR12-STi surfaces was slightly lower on day 3, consistent with the MTT results, and these cells exhibited a contracted and disjointed attachment morphology. Despite the initial observations of improper cell attachment on day 3, hFOB cells showed consistent growth on Sil-PTi and KR12-STi surfaces by day 7, with flattened morphology and increased cell spreading over time, indicating viability and proliferation. The number of attached cells on HELP-STi and KR12-HTi surfaces was notably higher than that on Sil-PTi and KR12-STi on day 3. These attached cells displayed extensive spreading by day 7, indicative of the excellent biocompatibility of the coating layer. By day 7, the surfaces of HELP-STi and KR12-HTi were fully covered by a thick layer of cells, with spread and flattened morphology and the formation of mineralized nodules suggesting cell-secreted mineralization and osteoblast differentiation.

Overall, normal cell attachment and proliferation on all surfaces highlight the excellent biocompatibility of both unmodified and modified titanium alloy surfaces. By day 7, the cells displayed spread morphology with filopodia extending toward adjacent cells, alongside the formation of mineralized deposits on KR12-STi, HELP-STi, and KR12-HTi surfaces (depicted by arrows in Figure 7b).

## 4 Conclusions

Preventing bacterial adhesion and biofilm formation while promoting osteointegration in medical implants is a significant challenge. This study explored a novel approach utilizing enzyme-responsive peptide-based coatings to address this issue. By harnessing enzyme-response mechanisms, a proof of concept is presented for the development of coatings with potent antibacterial properties, improved functionalities, and enhanced cytocompatibility. Specifically, the study investigated the potential of HELP-based matrixes as delivery systems triggered by enzymatic activity in the surrounding environment. During implantation, the customized interfaces encounter the innate immune response, triggering elastase release from the activated neutrophils, which promotes biodegradation of HELP biopolymers and enables the controlled release of AMPs.

Through experimentation, it was demonstrated that the AMP KR-12 can be effectively immobilized onto HELP-attached titanium surfaces, named KR12-HTi, at desired densities with high reaction yields. This coating exhibited excellent antimicrobial activity against clinically significant bacteria, including *S. aureus*, *S. epidermidis*, *E. coli*, and *P. aeruginosa*. Notably, this surface displayed the significant release of KR-12 upon exposure to elastase, indicating the high elastolytic degradation potential of HELP. Unlike traditional direct covalent immobilization methods, this coating was statically versatile, offering biocompatibility and cell adhesion without external stimuli.

The study highlighted the importance of intrinsic structural factors, such as linkers, surface density, and exposure orientation, in modulating the antibacterial activity of AMPs. The utilization of HELP as a linker resulted in a 3-fold increase in KR-12 loading. This enhancement can be attributed to the ability of the HELP linker to augment the number of anchors available for immobilization, thereby increasing the density of KR-12. Surfaces grafted with HELP exhibited higher antimicrobial activity against adhered bacteria, owing not only to the increase in loading density but also to the enhanced mobility and flexibility of KR-12 due to the elongated and flexible molecular structure of HELP.

The study highlighted the potential of stimuli-responsive peptide-based coatings to promote antimicrobial activity and osteoblast cytocompatibility. The potential to release KR-12 in response to increased elastase concentrations in the surrounding environment offers a promising strategy to mitigate common infections as well as promote bone formation through its effects on osteogenic differentiation. Finally, it is envisaged that the concourse of HELP as a linker could be implemented in new materials for a range of medical applications when drug release is required due to its double function to carry and then deliver the drug by the action of elastase enzymes.

## Supporting Information Available

Sequence of the HELP polypeptide, XPS survey spectra of modified titanium surfaces, calibration curve of dibenzofulvene-base adduct versus absorbance using 20% piperidine in DMF, and mechanism for Fmoc group removal from immobilized KR-12 (PDF)
