# Supplementary material for: Immobilization of KR-12 on a Titanium Alloy Surface Using Linking Arms Improves Antimicrobial Activity and Supports Osteoblast Cytocompatibility
Source: ACS Appl Bio Mater. 2025 Mar 28;8(4):2899–915. doi: 10.1021/acsabm.4c01731 (PMC12015957; doi:10.1021/acsabm.4c01731)
Supplement: Supplementary file 1 — mt4c01731_si_001.pdf [file mt4c01731_si_001.pdf]

# Supporting information

## Immobilisation of KR12 on a titanium alloy surface using linking arms improves antimicrobial activity and supports osteoblast cytocompatibility

Mohadeseh Zare [a*], Laura Colomina Alfaro [b], Antonella Bandiera [b], Esra Mutlu [a], David Grossin [c], Fernando Albericio [d], Sarah A. Kuehne [e], Zubair Ahmed [f], Artemis Stamboulis [a*].

[a] Biomaterials Research Group, School of Metallurgy and Materials, University of Birmingham, Edgbaston, Birmingham, B15 2TT, UK

[b] Department of Life Sciences, University of Trieste, via L. Giorgieri, 1, Trieste, 34127 Italy

[c] CIRIMAT, Toulouse INP, Université Toulouse 3 Paul Sabatier, CNRS, Université de Toulouse, 4 Allée Emile Monso - BP44362, 31030 Toulouse Cedex 4, France

[d] School of Chemistry and Physics, University of KwaZulu-Natal, Durban, 4000 South Africa

[e] School of Science and Technology, Nottingham Trent University, Nottingham, NG11 8NS, UK

[f] Neuroscience and Ophthalmology, Institute of Inflammation and Ageing, College of Medical and Dental Sciences, University of Birmingham, Edgbaston, Birmingham, B15 2TT, UK

*Corresponding authors: a.stamboullis@bham.ac.uk; zare.mohadeseh@gmail.com

### Details of the HELP polypeptide

HELP (clon. 22/3/2004) nel plasmide pEX8EL aa: 536 MW 44885.79 pI: 11.68

Sequence: MRGSHHHHHHGSAAAAAAAAKAAAKAAQFGL VPGVG VAPGVG VAPGVG VAPGVG LAPGVG VAPGVG VAPGVG VAPGIAPA AAAAAKAAAKAAQFGL VPGVG VAPGVG VAPGVG VAPGVG LAPGVG VAPGVG VAPGVG VAPGIAPA AAAAAKAAAKAAQFGL VPGVG VAPGVG VAPGVG VAPGVG LAPGVG VAPGVG VAPGVG VAPGIAPA AAAAAKAAAKAAQFGL VPGVG VAPGVG VAPGVG VAPGVG LAPGVG VAPGVG VAPGVG VAPGIAPA AAAAAKAAAKAAQFGL VPGVG VAPGVG VAPGVG VAPGVG LAPGVG VAPGVG VAPGVG VAPGIAPA AAAAAKAAAKAAQFGL VPGVG VAPGVG VAPGVG VAPGVG LAPGVG VAPGVG VAPGVG VAPGIAPA AAAAAKAAAKAAQFGL VPGVG VAPGVG VAPGVG VAPGVG LAPGVG VAPGVG VAPGVG VAPGIAPA AAAAAKAAAKAAQFGL VPGVG VAPGVG VAPGVG VAPGVG LAPGVG VAPGVG VAPGVG VAPGIAPGV

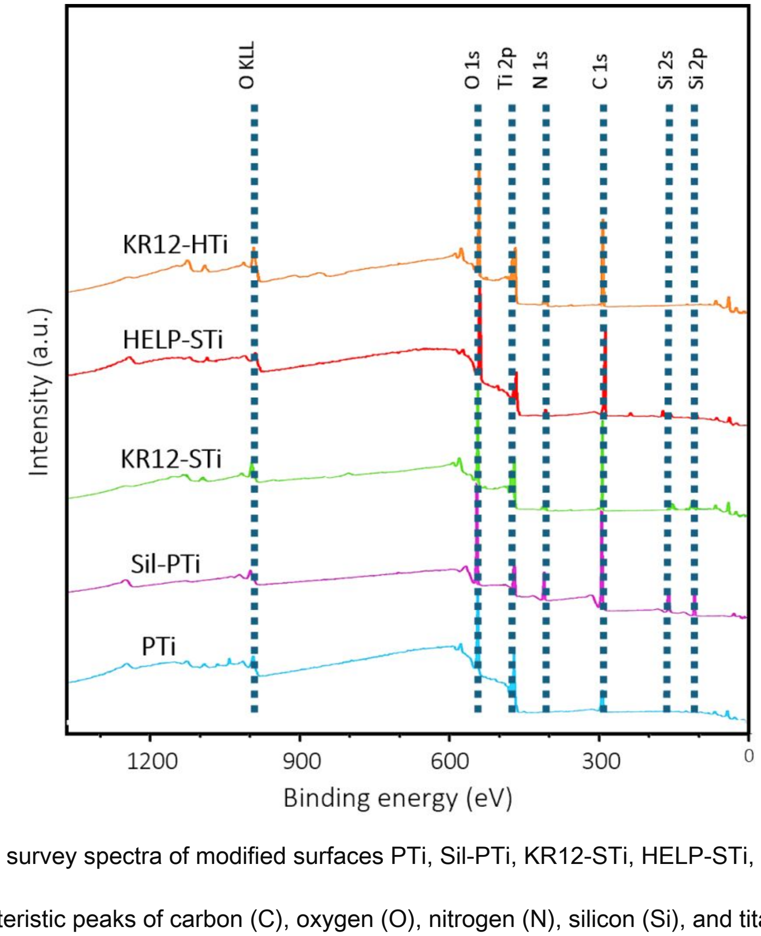


Figure S1. XPS survey spectra of modified surfaces PTi, Sil-PTi, KR12-STi, HELP-STi, and KR12-HTi with characteristic peaks of carbon (C), oxygen (O), nitrogen (N), silicon (Si), and titanium (Ti).

a)

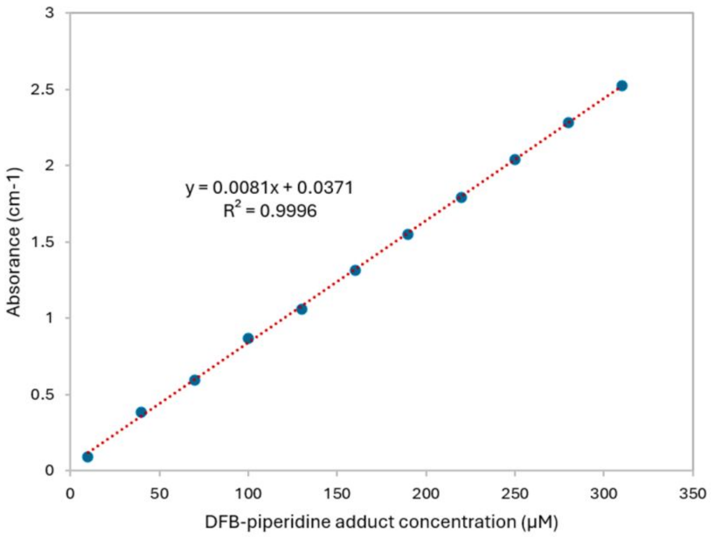


b)

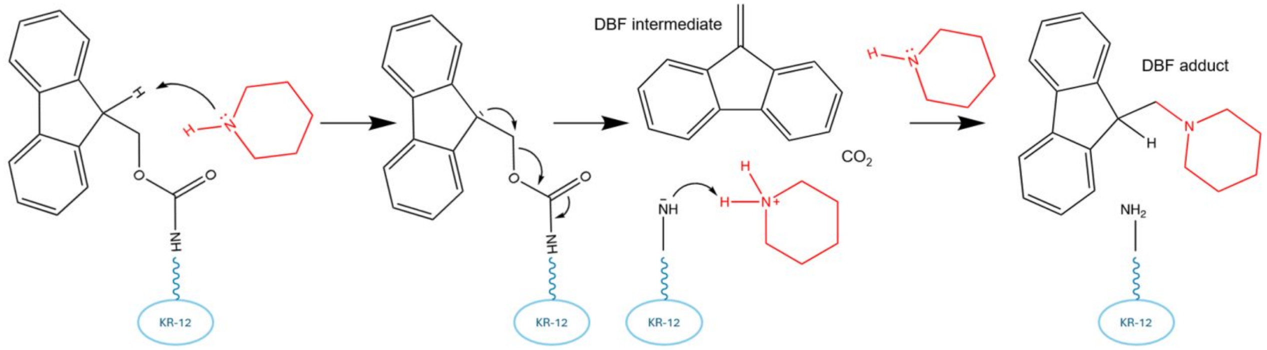


Figure S2. a) Calibration curve of dibenzofulvene-base adduct (µM) vs. absorbance, using 20% piperidine (Panel B) in DMF, b) Mechanism for Fmoc group removal from immobilized KR-12.
